# BioGlue^®^ is not associated with polypropylene suture breakage after aortic surgery

**DOI:** 10.3389/fsurg.2022.976944

**Published:** 2022-09-09

**Authors:** Davide Pacini, Giacomo Murana, David Hollinworth, William F. Northrup, Stacy G. Arnold, Roberto Di Bartolomeo

**Affiliations:** ^1^ Cardiac Surgery Unit, St. Orsola Hospital, University of Bologna, Bologna, Italy; ^2^ Division of Cardiac Surgery, Cardiac Surgery Department, IRCCS, Azienda Ospedaliero-Universitaria di Bologna; ^3^ Department of Experimental, Diagnostic and Specialty Medicine, DIMES, University of Bologna, Bologna; ^4^ Artivion Inc., Guildford, United Kingdom; ^5^ Artivion Inc., Kennesaw, GA, United States

**Keywords:** BioGlue^®^, polypropylene suture, suture breakage, aortic anastomosis, cardiovascular surgery, *in vitro* **testing**

## Abstract

**Objective:**

We have encountered broken or damaged polypropylene sutures (Prolene^®^) at the anastomotic sites during aortic reoperations. Because a surgical sealant, bovine serum albumin-glutaraldehyde (BioGlue^®^), was used in previous aortic surgery in some of these cases, we undertook this *in vitro* study to evaluate whether the use of BioGlue^®^ was associated with breakage of polypropylene sutures at the aortic anastomosis.

**Materials and methods:**

The broken polypropylene sutures, anastomotic sites and aortic tissue at the location of suture breakage were visually inspected and evaluated intraoperatively. Six human cadaveric aortic samples were incised circumferentially and anastomosed proximally to a valved conduit with running 4–0 polypropylene sutures (Prolene^®^). In the test group (*n* = 3), BioGlue^®^ was applied directly to the Prolene^®^ sutures at the anastomotic sites, while in the control group (*n* = 3) the anastomoses were not sealed with any surgical adhesive. The six samples were immersed in Dulbecco's phosphate buffered saline solution and mounted on a M-6 Six Position Heart Valve Durability Testing System and tested up to 120 million cycles for a 2-year period*.* During and upon completion of the testing, the integrity of Prolene^®^ sutures, the anastomosis and aortic tissues was regularly assessed by visual inspection.

**Results:**

Intraoperative findings included a stretched and thin aortic wall (some with thrombus), a small cleft between the aortic tissue and the Dacron vascular graft. An excessive amount of BioGlue^®^ was often found around the anastomosis, with cracking material, but no signs of mechanical damage were observed in these cases. Upon visual inspection during and after *in vitro* testing, there was no apparent damage to the polypropylene sutures on the interior or exterior of the aortic anastomoses in any of the samples. No difference was observed in the physical integrity of the polypropylene sutures at anastomotic lines, the anastomoses and aortic tissues between the test and control samples.

**Conclusions:**

The results of this study suggest that the use of BioGlue^®^ was not associated with breakage of the polypropylene sutures at the anastomotic sites after aortic dissection repair.

## Introduction

In aortic dissection repair, it is vital to ensure good sealing around the anastomotic suture line and uniform reapproximation of the dissected aortic layers to avoid problematic postoperative bleeding and minimize the need for late reintervention (1). With the development of surgical adhesives or sealants, it has become a common practice in many centers to seal the anastomotic suture lines with a surgical adhesive (2, 3). In 2001, bovine serum albumin-glutaraldehyde (BSAG, BioGlue®, Artivion, Kennesaw, GA) was approved by FDA in 2001 to improve hemostasis of suture lines and reinforce the fragile aortic tissue during acute dissection repair (4). It has been extensively used in various clinical settings and shown to be a safe and useful adjunct in aortic surgical procedures with appropriate use (5–7).

**Figure 1:**
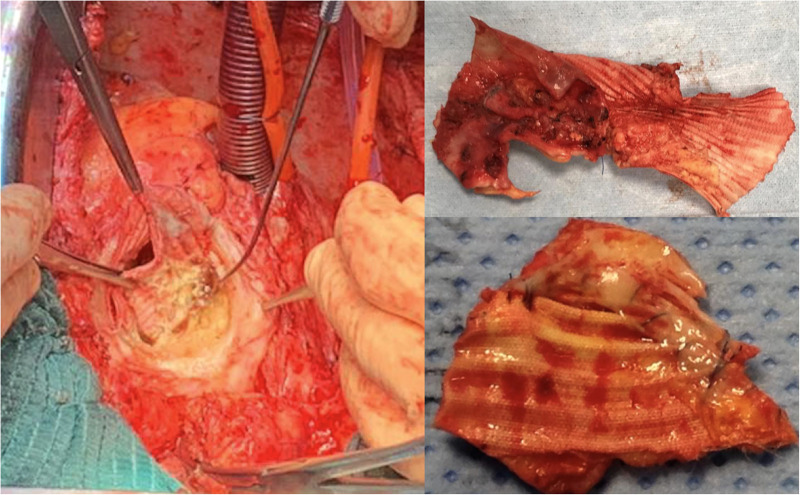
Intraoperative findings of damaged polypropylene suture and discontinuity between the aortic wall and the vascular graft at the proximal anastomotic site where BioGlue^®^ was used in a patient undergoing prior aortic dissection repair.

At the University of Bologna, we have been using BioGlue® as an adjunct in surgical repair of aortic dissections to reapproximate the dissected intimal layers. During reoperation on patients undergoing prior aortic dissection repair, we have found damaged or broken polypropylene sutures at the anastomotic site occasionally. Among 100 aortic reoperations performed over a 2-year period, broken polypropylene sutures were observed in 4 patients at the anastomotic sites where BioGlue® had been used during prior aortic surgery (Figure 1). Visual inspection of these broken sutures did not reveal any signs of mechanical damage that may be ascribed to the use of BioGlue®. Nor did an exhaustive literature search find any published reports of suture breakage related to the use of BioGlue® after cardiothoracic and vascular surgery. Up to date, only one study reported breakage of absorbable 6–0 polydioxanone (PDS) or 6–0 polyglactin 910 (Vicryl) sutures in children with hypospadias for whom BioGlue® was used to reconstruct the urethra (8). This prompted an institutional review on the use of surgical sealants in aortic dissection repair, and, as a precautionary measure, a decision was made to suspend the use of surgical adhesives or sealants during aortic operations at our hospital.

In order to determine if the use of BioGlue® contributes to the breakage or failure of polypropylene sutures (and potential mechanisms for interaction), we discussed our observations with the research team at CryoLife Inc (now Artivion), manufacturer of BioGlue®. Artivion has not received any complaints of polypropylene suture breakage or damage caused by BioGlue® since 2001. Consequently, Artivion invited our team to perform a study to assess whether BioGlue® interacts with or has some mechanical impact on the physical integrity of polypropylene sutures. The purpose of this study was to evaluate whether the use of BioGlue® was associated with breakage of the polypropylene sutures at the anastomotic sites following aortic dissection repair.

## Materials and methods

The Research Ethics Committee of the University of Bologna approved this *in vitro* study. The investigation was carried out strictly in accordance with our study protocol by the research team at Artivion's laboratories in Kennesaw, Georgia, USA, where we have visited to ensure that the execution and quality standards of the investigation match our requirements.

The study was designed to simulate the lateral and longitudinal mechanical movements of the human aorta *in situ* following circumferential aortic anastomosis with the suturing material and technique, and the application of BioGlue®, as would be performed at our hospital.

### Intraoperative visual inspection

The broken polypropylene sutures, the anastomotic sites and the aortic tissue at the location of suture breakage were visually inspected during the reoperation, evaluated and recorded, respectively.

### Aortic tissue preparation

This study was conducted using six human cadaveric aortic valves and ascending aorta, each having received donor consent. The six aortic samples were divided equally into the test and the control groups. All six samples received the same treatment, except that BioGlue was applied to the aortic anastomotic lines in the test group.

**Figure 2:**
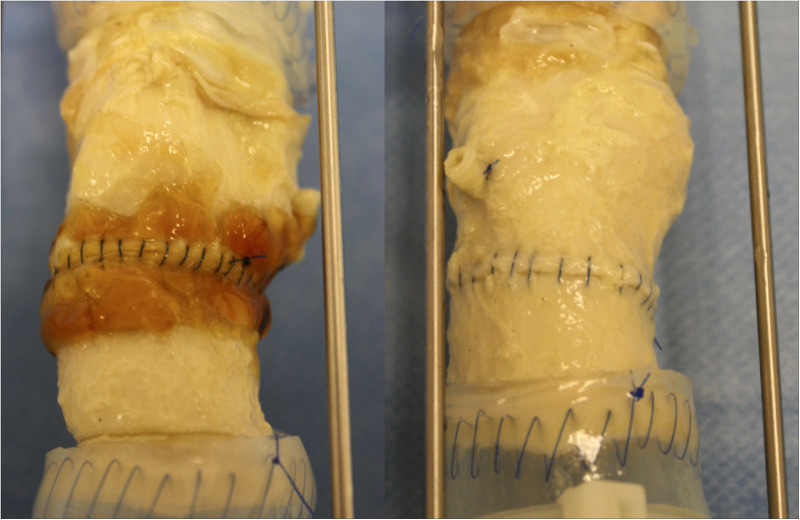
Images of the anastomotic suture lines of the test (left) and control (right) groups at 120 million cycles.

The ascending aorta was circumferentially transected 1 cm above the sinotubular junction and then re-connected proximally to a valved aortic conduit, CryoValve® Aortic Allograft (Artivion, Kennesaw, GA) with a running 4–0 polypropylene suture, Prolene® (Ethicon, Somerville, NJ). After completion of the aortic anastomoses, 2–3 ml of BioGlue® was applied to the anastomotic suture line of three samples in the test group in compliance with the Instructions for Use approved by FDA (9). BioGlue® appears as a thin, translucent orange-brown layer around the anastomotic suture line (Figure 2, left panel). The anastomotic suture lines in the three samples of the control group were not sealed with any surgical adhesive or sealant.

### Physiological simulation and durability testing

**Figure 3:**
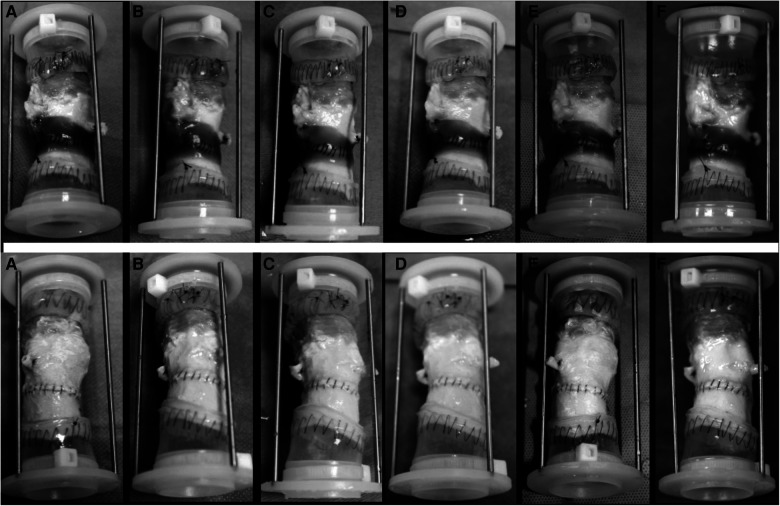
Images of the test group (upper row) and the control group (down) at 20 million cycle intervals. (**A**) 20 million cycles; (**B**) 40 million cycles; (**C**) 60 million cycles; (**D**) 80 million cycles; (**E**) 100 million cycles, and (**F**) 120 million cycles.

Each anastomosed aortic sample was fitted onto a universal valve mount, which was loaded into a valve chamber on an M-6 Six Position Heart Valve Durability Testing System (Dynatek Labs, Galena, MO) (10, 11). The samples within the testing system were immersed and maintained in Dulbecco's phosphate-buffered saline (DPBS) solution (Thermo Fisher Scientific, Waltham, MA) at body temperature throughout the testing period (Figure 3).

The testing system was set to 200 cycles per minute with a constant transvalvular pressure (closure load) of 100 mmHg across each aortic valve. The cycle rate and closure load were selected in compliance with the ISO Standard 5,840 Guidelines for quasi-real-time testing of viscoelastic materials (12). The minimum aortic peak differential pressure was monitored weekly to ensure that constant pressure be maintained throughout the testing period. The testing was continued to a total of 120 million cycles, which simulates the physiological functioning of the natural aorta and aortic valve in the human body for a two-year period.

### Monitoring and assessment

The aortic samples were visually inspected daily to assess the condition and integrity of the Prolene® sutures, the anastomotic site, the aortic tissue and the aortic valve. A strobe light was used to simulate a static visual field enabling inspection of the samples while the high-speed cycling continued without interruption. After every 20 million cycles and at 120 million cycles, the samples were removed from the equipment and visually inspected. The appearance of the polypropylene sutures, the aortic tissues and anastomoses was recorded with photographs and a written description of the findings. The observers were randomized and double blinded to the study.

## Results

### Intraoperative findings

Intraoperative inspection showed a stretched and thin aortic wall (like that of a pseudoaneurysm), sometimes including thrombus material as a result of a localized inflammatory reaction. A common finding was a small cleft between the aortic tissue and the Dacron vascular graft in relationship to the old sutures or due to the progression of aortic dilatation. In the areas around the anastomosis an excessive amount of BioGlue® was often found, with cracking material which is compatible with degradation of the glue, but no apparent signs of mechanical damage were observed in these cases.

### *In vitro* testing

**Figure 4:**
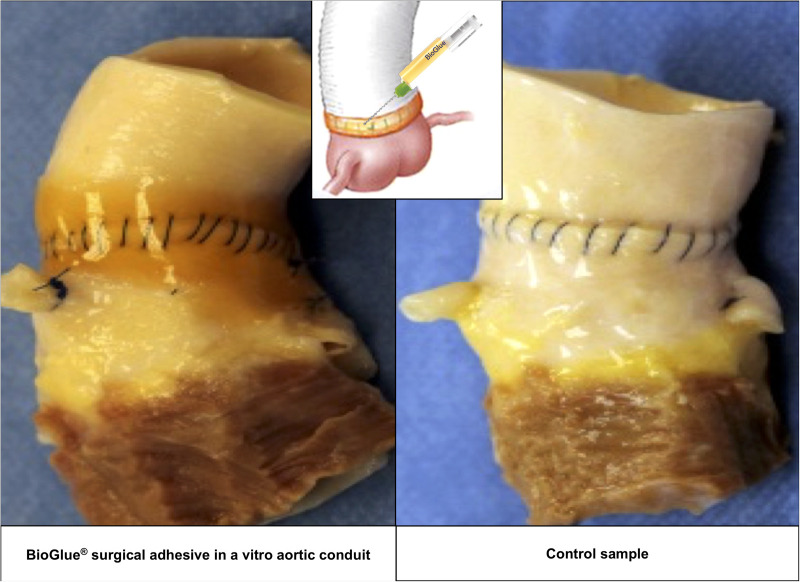
Aortic conduits in the BioGlue^®^ group (left) and the control group (right).

**Figure 5:**
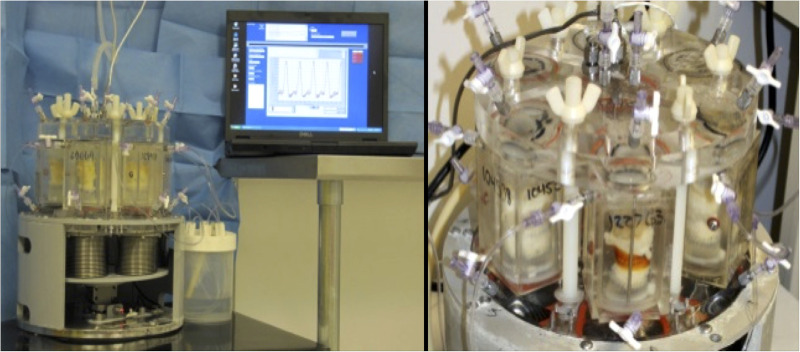
Aortic conduits mounted on M-6 Six Position Heart Valve Durability Testing System.

All aortic samples and CryoValve® aortic allografts were functioning well to the completion of the *in vitro* testing. Images of the test and control samples at six evenly spaced intervals and at 120 million cycles are shown in Figures 4, 5 shows the images of the test and control samples upon completion of this study.

Upon close visual inspection, there was no visibly apparent damage to the polypropylene sutures at the aortic suture lines – both inside and outside each valved aortic conduit – at each of the five 20-million cycle intervals and at the end of the testing upon completion of 120 million cycles. The polypropylene sutures remained intact in all samples and no difference was found in the physical integrity of the Prolene® sutures between the samples of the test and control groups.

Cracking of the BioGlue® was observed visually in one sample at the end of the study, which did not impact the suture lines upon visual inspection.

Upon visual examination, the aortic tissues around the anastomotic suture line and valve conduits were normal and did not reveal any signs of dehiscence, tearing or other abnormalities in the aorta.

## Discussion

The results of this *in vitro* study show that 4–0 polypropylene sutures covered with a thin layer of BioGlue® at the anastomotic suture line remained intact during *in vitro* testing conditions that simulated two years of lateral and longitudinal movement of the anastomosed aortic tissue in the human body. This investigation was carried out in conditions that resemble the clinical scenario and *in vivo* setting in which BioGlue® is utilized to reinforce the suture lines of aortic anastomosis. The use of Prolene® sutures and continuous stitches are the standard practice in aortic repair at the University of Bologna. The M-6 Six Position Heart Valve Durability Testing System used in this study has previously been reported to be an effective *in vitro* measure of the mechanical characteristic of materials, fatigue life, and fluid dynamic performance and representative of *in vivo* physiological conditions (10, 11). Although less akin to blood than Hank's balanced salt solution, the DPBS solution simulates isotonic physiological conditions and its transparency allows for easy visualization of the anastomotic suture lines during the testing period.

Upon completion of the *in vitro* testing, there was no visible difference in the physical integrity of the polypropylene sutures in the aortic samples treated with BioGlue® and in control samples without treatment of BioGlue®. The observed cracking of BioGlue® is reflective of the known mechanical degradation process of this adhesive over time. The lack of abnormality in the aortic tissue around the anastomotic suture line at the end of the testing implies that the application of BioGlue® did not affect the aorta in a way that might impede the free movement of sutures. These results imply that BioGlue® does not cause mechanical damage to the polypropylene sutures used to seal the anastomosis or reapproximate the dissected layers during aortic dissection repair. Although only Prolene® sutures were tested in this study, the implications of this study may apply to polypropylene sutures in general (13), including Surgipro™ II (14), given that their properties and tensile strength have been shown to be largely comparable.

While the results of this study suggest that the use of BioGlue® did not contribute to the failure of sutures at the aortic anastomosis, the question remains to be answered as to how the polypropylene sutures were damaged or broken. In cardiovascular surgery, nonabsorbable, synthetic monofilaments have been the standard material for tissue-to-tissue and prosthetic-to-tissue anastomosis. Since its invention in the 1960s (15), the polypropylene suture has been a well-established monofilament with very good physical, chemical and mechanical resistances as well as ease of handling. Its merits include excellent maintenance of tensile strength with no biodegradation, low coefficient of friction with less tissue trauma, low tissue reaction, low thrombogenicity, less chance of infection, and less intraoperative blood loss (16). It can maintain a high degree of biological and chemical inertness after long periods in tissues, which implies minimal likelihood of chemical interactions between polypropylene and BioGlue® or tissue (17).

Suture and knot failure have been implicated in 12% of abdominal wound dehiscence (18) and in 1.4% after aortic valve repair (19), however, the true incidence of polypropylene suture breakage or fracture after cardiac, aortic and vascular operations remains unknown. This problem has been reported only sporadically and experience is confined to case series or small cohorts (19–28). In a review on aortic valve repair, Carr and Savage found the incidence of suture line dehiscence was 1.4% (11 of 761), but the types of suture material were not specified. In literature, the surgical procedures included mitral valve replacement (22, 23, 26), aortic valve repair (19), closure of atrial septal defect (20, 21) or patent ductus arteriosus (27), coronary artery bypass grafting (26), femoro-popliteal bypass (24, 27), and ascending aortic replacement (25). Suture breakage or rupture may occur as early as intraoperatively or in the immediate postoperative period, or as late as 5.3 years after surgery (19), leading to severe bleeding, failed valve repair (19), periprosthetic leakage (26), or anastomotic false aneurysm (24). The sizes of broken sutures ranged from 5–0, 4–0 to 2–0, with 5–0 being the most common. The breakage may be located at the atrial septal defect (20, 21), aorto-to-coronary graft anastomosis (27), valve annulus (19, 22), aortotomy (27), arteriotomy (24), left atriotomy (23), graft-to-graft anastomosis (24), or distal aortic anastomosis (25), either close (at the base of) or at some distance to the knots.

Although the exact mechanisms remain largely unclear, various factors from production and packaging process to intraoperative handling may cause or contribute to the breakage or fracture of polypropylene sutures after cardiovascular surgery (24, 25, 29). In an analysis of suture fracture morphology, Karaca and Hockenberber found that the polymer type, size of suture, and knot security played important roles in the breaking process (30). The reliability and security of knots have been repeatedly shown to affect the geometry and tensile strength of sutures (31). Aanning and colleagues found that Prolene® sutures anchored with square knots retained only 75% of their strength compared with half hitches (32), while running 3–0 monofilament and multifilament sutures anchored with square knots retained only 50% to 84% of the strength of identical sutures secured with half hitches (33). These findings show the impact of knot types on suture security and suggest that running polypropylene suture anchored with half hitches is stronger and safer than the same suture secured with square knots.

An experimental study (34) examining the effects of surgical manipulation on suture tensile strength reveals that pulling sutures over the torn edge of the foil package, permanently kinking sutures, or axially twisting them up to 4 times did not decrease tensile strength; nor did the tug exerted by the scrub nurse on polypropylene sutures decrease their tensile strength. However, the presence of a stray knot reduced suture strength by 17%, and grasping sutures with forceps decreased the suture strength in a dose-dependent fashion. These results imply that polypropylene sutures with a stray knot should be discarded and instrumentation of sutures with needle holders, forceps or clamps should be avoided (34). Thermal damage to polypropylene sutures from electrocautery was also found to be a risk factor for suture rupture, even with very short contact time (25). In patients with late anastomotic pseudoaneurysm after vascular (graft)-to-graft anastomosis, the mechanisms of suture fracture include the sawing effect of the rigid structure(s) on the suture line during each arterial pulsation, chronic loading secondary to hypertension (28, 35), and, possibly, heavy calcification of the arteries (or aorta) (24).

As far as the broken sutures we encountered are concerned, it is possible that the sutures may have been weakened or damaged by excessive pressure applied by surgical devices or accidental introduction of knots in the filament during the suturing process (34). The occurrence of these suture failures is most likely to be multifactorial and may not be possible to elucidate experimentally given the uniqueness of each patient's interior milieu and pathophysiology, as well as the diversity of surgeon experience and technical proficiency while performing the initial aortic procedures. Although Azadani and associates have found that BioGlue has higher stiffness and less compliance compared to other surgical sealants in an experimental study (36), which leads to shearing effects during each cardiac cycle and may generate excess stress on the tissue or aortic suture line, the results of our *in vitro* testing suggest that the likelihood of BioGlue® causing the suture breakage is very low. In addition, some studies on repair of postinfarction ventricular septal defect also showed that BioGlue® may strengthen the suture lines and decrease the likelihood of suture dehiscence (37, 38).

On the basis of the present study and literature review on BioGlue®, we have resumed the use of BioGlue® as a surgical sealant during aortic surgery in our hospital. As with all adjuncts used in surgical procedures, it is imperative that BioGlue® be used with utmost care and precision to avoid complications (39). In addition to strictly following the instructions for use by applying BioGlue® as little as possible in a dry field, we apply it only on the outside of the anastomotic suture line and do not use BioGlue® to reapproximate the dissected aortic layers (9), which conforms to the judicious advice that “caution must be taken to avoid using excessive glue” (39).

### Study limitations

This study is limited by the small number of study samples, and the assessment of the suture integrity by visual rather than microscopy, fluorescence, and leak tests, which precludes quantitative measures to evaluate anastomotic compromise and suture integrity, despite that our recent lab testing showed BioGlue® had a shear strength of 2,488.3 ± 126.4 gf/cm^2^ (range 2,136–2,734). Based on our experience and literature review, the potential incidence of BioGlue®-related suture breakage is so low (possibly much lower 0.1%) that this study may be underpowered to detect any effect of the BioGlue®. Other concerns pertain to the use of an *in vitro* testing system with aortic samples immersed in DPBS solution, which is less representative of the complex physiological milieu compared to an *in vivo* system immersed in blood or other solutions more akin to blood (such as the Hank's balanced salt solution). Neither did we evaluate the potential direct chemical interaction between BioGlue® and polypropylene. This will be the focus of our further study, which includes the chemical reaction of the two entities in the immediate phase as well as over a long period of time. For these reasons, we interpret the results of this study as suggestive of no mechanical interaction between BioGlue® and polypropylene sutures, with very little chance of BioGlue® causing the suture breakage. Further post-market monitoring and experimental testing are warranted to elucidate the exact cause and mechanism of suture breakage at the aortic anastomosis.

## Conclusions

The results of this study suggest that the use of BioGlue® was not associated with mechanical damage or breakage of polypropylene sutures at the anastomotic sites after surgical repair of aortic dissection.

## Data Availability

The raw data supporting the conclusions of this article will be made available by the authors, without undue reservation.
